# The gut-derived metabolites as mediators of the effect of healthy nutrition on the brain

**DOI:** 10.3389/fnut.2023.1155533

**Published:** 2023-06-09

**Authors:** Quentin Leyrolle, Lucia Prado-Perez, Sophie Layé

**Affiliations:** NutriNeurO, UMR 1286, Bordeaux INP, INRAE, University of Bordeaux, Bordeaux, France

**Keywords:** nutrition, gut-brain axis, gut-derived metabolites, behavior, fibers, polyphenols, polyunsaturated fatty acids, postbiotic

## Abstract

Nutrition is now well recognized to be an environmental factor which positively or negatively influences the risk to develop neurological and psychiatric disorders. The gut microbiota has recently been shown to be an important actor mediating the relationship between environmental factors, including nutrition, and brain function. While its composition has been widely studied and associated with the risk of brain diseases, the mechanisms underlying the relationship between the gut and brain diseases remain to be explored. The wide range of bioactive molecules produced by the gut microbiota, called gut-derived metabolites (GDM), represent new players in the gut to brain interactions and become interesting target to promote brain health. The aim of this narrative review is to highlight some GDMs of interest that are produced in response to healthy food consumption and to summarize what is known about their potential effects on brain function. Overall, GDMs represent future useful biomarkers for the development of personalized nutrition. Indeed, their quantification after nutritional interventions is a useful tool to determine individuals’ ability to produce microbiota-derived bioactive compounds upon consumption of specific food or nutrients. Moreover, GDMs represent also a new therapeutic approach to counteract the lack of response to conventional nutritional interventions.

## 1. Introduction

The influence of dietary habits on the incidence of non-communicable brain diseases such as psychiatric and neurodegenerative disorders, has been extensively reported by observational studies in humans (1–3). Regular consumption of foods rich in saturated fats and/or carbohydrates as well as ultra-processed foods and meat has been highlighted as potentially harmful for brain health. On the other side, regular consumption of vegetables, fruits and/or fish characteristic of Mediterranean (Medi-diet), “Dietary Approaches to Stopping Hypertension” (DASH) or Mediterranean-DASH Intervention for Neurodegenerative Delay (MIND) diets, has been associated with beneficial effects (4–6). The positive effects of these diets on the brain could be due to the neurobiological effects of specific nutrients/micronutrients contained in these foods. Several clinical and preclinical studies indicate that n-3 polyunsaturated fatty acids (PUFAs), polyphenols, and dietary fibers, which are present in fatty fish, fruits, and vegetables, have neuroprotective effects (7–9). In addition, B vitamins which are present in green leafy vegetables (an important component of the MIND diet), have been associated with a lower risk of dementia or aging-related brain atrophy (10, 11). Overall, insufficient vitamins intake has been associated with increased risk for neuropsychiatric disorders or cognitive decline (12–14). However, the neurobiological mechanisms underlying the individual and/or added effects of these nutrients/micronutrients are still poorly understood, making translation to humans difficult. The clinical trials aiming at using dietary intervention (food or nutrients/micronutrients alone or in combination) in the management of brain diseases shows that there are substantial variations in the response of individuals to these dietary interventions (15). Here, it is considered that a responder to a dietary intervention is characterized by the improvement of mental health and/or brain functioning. This improvement is biologically defined by a specific GDM/CM profile in responder that is distinct to the one of non-responders who do not have brain health improvement. Individual response to food is influenced by physiological factors, such as gender and age, by metabolic factors such as weight status and exercise, and by genetics. It is important to mention that other behaviors, such as the practice of physical activity and contextual factors, are also key in this diversity of response (16, 17). However, these aspects have not been really taken into account for the understanding of the relationship between diet and brain health which is crucial to establish dietary recommendations adapted to the individuals. A novel important actor participating to the individual response to food is the gut microbiota. Indeed, the latter turns out to be not only influenced by diet but also to influence host’s health (18, 19). Recent reports indicate that the interaction between the intestinal microbiota and the individual’s physiology could possibly influence brain health (20). These nutrition-microbiota-host physiology interactions lead to the production of a wide range of molecules that reach the different organs through the bloodstream including the brain. Thus, distinct individual neurobiological response to food could depend on the ability to produce these gut-derived molecules.

The circulating metabolome contains molecules directly derived from food (minerals, some vitamins) and from the digestion of such food by the gut microbiota. These latter are termed gut-derived metabolites (GDM) and include several type of molecules like bioactive lipids, bile acids, short-chain fatty acids (SCFA), phenolic compounds and neurotransmitters (21). These GDM can give rise to phase II metabolites or co-metabolites (CM) under the metabolization of host enzymes like glucuronidation, sulfatation, methylation. 58% of blood metabolites variability can be explained by the gut microbiota composition (22). Bar et al. (23), have elegantly shown that gut microbiota composition, dietary habits as well as the clinical profile are major factors influencing the circulating metabolome. Deciphering the respective contribution of nutrition, gut microbiota and the subsequent metabolic and inflammatory conditions on brain health is crucial to adapt nutrition-based interventions to individuals. Recent clinical studies addressed this important question. Indeed, the potential causal link between gut microbiota disturbances and neurodevelopmental disorders (like autism) (24–26) has been challenged by a recent study from Yap et al. (27). In this study conducted in autistic children, authors revealed that the restricted diet adopted by the patients, rather than a shift in gut microbiota composition, is predictive of the symptoms (27). This illustrate the importance of considering not only gut microbiota composition but also dietary habits as well as clinical profile and the GDM/CM circulating signature of patients suffering of brain diseases. This is crucial to design efficient nutritional or gut microbiota targeting interventions.

The aim of this scoping review is to summarize current knowledge on the potential role of GDM and CM as biological mediators of the effect of nutrition and gut microbiota on brain health. Indeed, we bring in this review a collection of selected data from the literature to feed the new concept that a part of the effect of nutrition on brain function relies on these bioactive compounds. In addition, we bring new angles of data interpretation to feed the concept that molecular mechanisms underlying the inter-individual differences in the response towards nutrition-based interventions at the behavioral level. First, we will present some diets and food components that have been shown to be protective against brain disorders. Then, we will discuss the possible role of GDM and CM in mediating the beneficial effects of above-mentioned diets on brain function.

## 2. Diets promoting brain health

Several types of healthy diets have been associated with the promotion of brain health and the protection toward neuropsychiatric and neurological diseases. These diets include plant-based diet, the Medi-diet, the DASH and the diet. These diets follow some of the world health organization (WHO) eating guidelines that advice to increase consumption of vegetables, fruits, whole grain and dietary fibers while decreasing consumptions of fats, sugars and salt (28). The Medi and MIND diets have been shown to improve cognition and morphological brain parameters, such as cortical thickness and white matter integrity in healthy elderly or inferior frontal gyrus surface in obese women (29–31). In normal aging process, the consumption of green Medi-diet, which consist in a Medi-diet coupled to a supplementation on two sources polyphenols (green tea and *Wolffia globosa* duckweed also called “Mankai”) has been shown to slow the age-related brain atrophy (32). Greater adherence to a dietary pattern consistent with a plant-based diet was related to better performance on all cognitive tasks in older adults (33). The anti-inflammatory and antioxidant properties of some foods present in these diets have been proposed to be the biological contributors of their brain health-promoting effects (3, 33, 34). In addition to its pro-cognitive activity, the Medi-diet is associated with a decreased risk of developing mood disorder in adults and elderly as shown by several observational studies (35–41). Moreover, the Medi-diet alleviated depression risks or symptoms in two interventional studies (42, 43) while another intervention based on dietary advice resulting in increased consumption of fibers and polyunsaturated fatty acids (PUFAs) led to a decrease in perceived stress (44). Of note, two studies highlighted that the protective role of Medi-diet against depression is noticeable in cross sectional studies while it is less clear in longitudinal studies warranting more studies to understand the short *vs* long-term impact of Medi-diet adherence (45, 46). From all the aforementioned studies it appears that diet quality (intake of fruits, vegetables, fish, whole grain) may have a protective role towards neurological and psychiatric disorders (47, 48).

**Figure 1:**
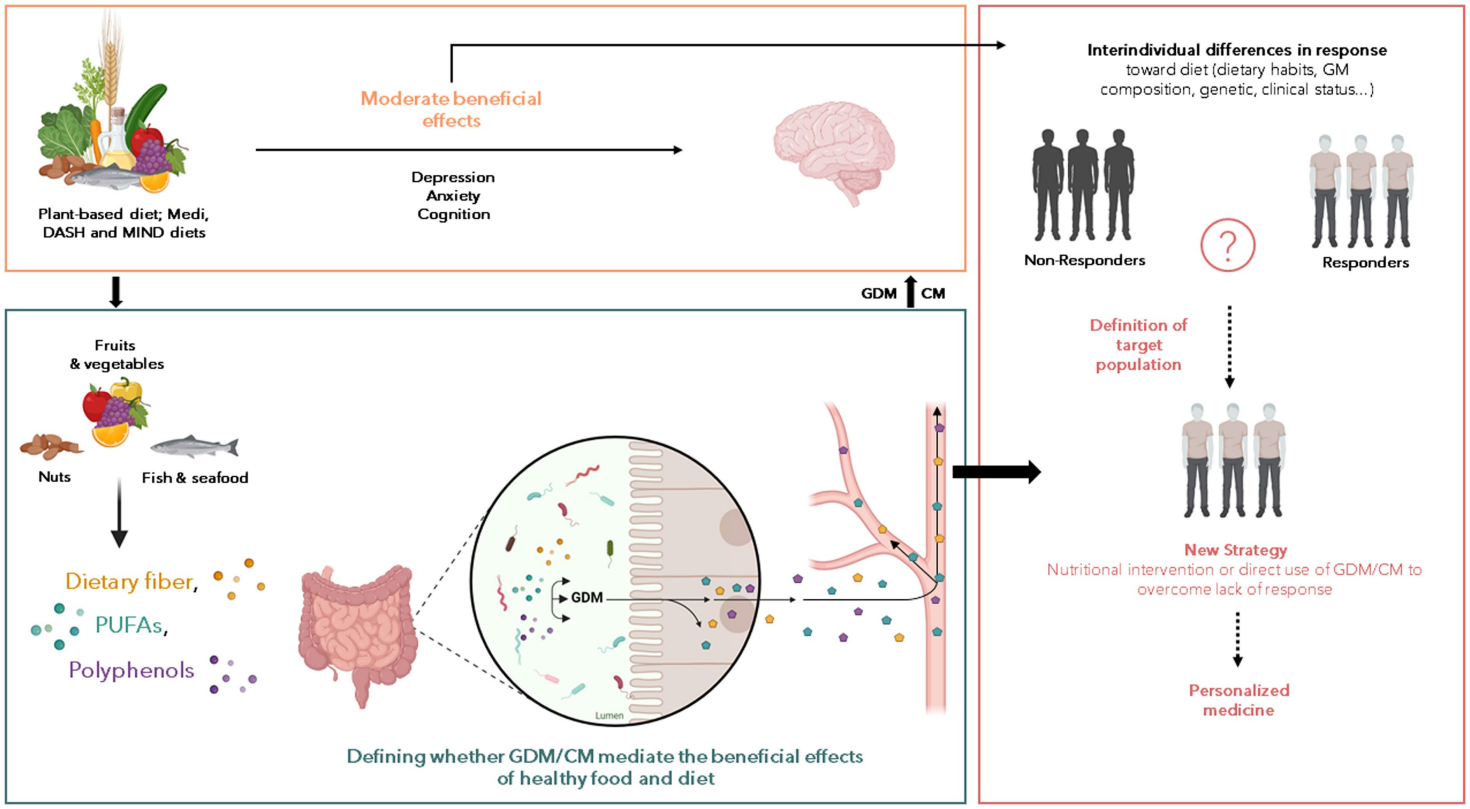
Potential of gut-derived metabolites (GDM) and co-metabolites (CM) to improve the effect of nutritional intervention on the neurological and psychiatric disorders. Numerous reports of associations between nutrition and beneficial or harmful consequences on the brain have been published over the last decades but the nutritional interventions displayed limited effects. Several factors interact with each other’s to influence the response toward food intervention including the gut microbiota composition, genetic and clinical status and we postulate that the GDM and CM production from food can be a pivotal factor explaining such differences. Thus, the understanding of factors that characterize the positive response toward nutritional intervention is crucial to (1) decipher the role of GDM and CM in the beneficial effect of healthy food on brain health, and (2) to define population to target for nutritional intervention aiming to promote brain health. The so-called “non-responders” could benefit from intervention based on GDM or CM. These approaches can be combined with medications, pre or probiotics to achieve a synergistic effect. But the main goal here is to being able to understand and bypass the “resistance” to nutritional intervention to define personalized strategy according to clinical profile of subjects.

However, the large variability in the response towards nutritional interventions is a potential limitation in the use of dietary interventions to protect and/or counteract brain disorders. It is therefore crucial to better understand the parameters which define the response of individual as it could on several parameters that remain poorly characterized. The gut microbiota function (i.e.:, its ability to process foods and to produce a wide range of bioactive molecules) is an interesting target to better understand this variability. Even if out of the scope of this review several other parameters can be important in the inter-individual variability towards nutritional interventions. They include genetic factors (49), metabolic health or underlying conditions like diabetes or kidney diseases (50–52). The production of GDM or CM from several types of nutrients highlighted in the previous part has been shown to be largely variable between individuals (53–56). This is notably the case for the production of the bioactive derivatives of polyphenols (53, 57, 58). For example, the association between coffee consumption and a lower dementia risk in elderly is affected by inter-individual differences in coffee metabolism (49), which has been shown to be influenced by gut microbiota composition and activity (59). Also urolithin A, a GDM from polyphenols, is produced by less than half of those consuming ellagic acid found in berries and walnuts (54). In response to Flavan-3-ol consumption (enriched in coffee and cocoa), three different populations or “metabotypes” have been reported based on the presence of the metabolites trihydroxyphenyl-γ-valerolactones, dihydroxyphenyl-γ-valerolactones (DPVL) or hydroxyphenylpropionic acids (60, 61). The pivotal role of the gut microbiota in these metabotypes has been highlighted and some bacteria has been found to be responsible for the ability to produce these polyphenol-derived GDM (62). Inter-individual differences in the response toward other food or nutrients of special interest for brain health has been described. For example, the prebiotic effect of different dietary fibers, measured by assessing the changes in the production of the short chain fatty acids (SCFAs), is controlled by the gut microbiota and the habitual dietary fiber intake (63). Moreover, the emotional improvements in response to dietary fiber intervention in obese subjects has been shown to be associated with differences in the prior gut microbiota composition (15) further illustrating the difficulties to predict the response to a dietary intervention. One actual challenge in human nutrition is to identify relevant biomarkers predictive of a positive response to nutritional intervention for the promotion of brain health (Figure 1). Besides this objective, the use of GDM or CM to overcome the inter-individual variability in nutritional intervention is appealing. Indeed, these compounds, alone and in combination, can be responsible for beneficial effects of healthy food in preventing neuropsychiatric and neurological illnesses. Thus, observational studies help to identify potential GDM or CM candidates while preclinical and clinical intervention allow to test their potential protective effects in different neuropathological conditions.

## 3. Gut-derived metabolites as mediators of healthy food effects on the brain

In this section, GDM and CM produced in response to fibers, polyphenols and PUFAs and their brain effects will be presented (Table 1). The focus on these GDM and CM relies on the fact that their parent nutrients are enriched in fruits, vegetables or fish which consumption is associated with beneficial effects on brain pathological processes both in preclinical and clinical settings (7, 125, 126). GDM that are not derived from the aforementioned nutrients will not be included in this review despite potent action on brain. GDM that are not derived from the aforementioned nutrients will not be included in this review despite potential effect on brain functions. For example, in the context of aging and dementia it has been shown that disturbances of the vasculature and BBB integrity can promote pathological processes (127). Recently, trimethylamine N-oxide (TMAO) or the p-cresol glucuronide, two GDM originating from choline and tyrosine respectively, has been shown to promote BBB function (128, 129) while at the opposite the p-cresol was associated with several detrimental effects such as inducing autistic-like behavior or impairing synaptic function in preclinical models (130, 131).

**Table 1:** Gut-derived metabolites, their precursors and their effects both in the CNS and at the periphery.

Metabolites	SCFA	DPVL	DHCA	Hippurate
Food sources	Fibers	Catechins and epicatechins	Chlorogenic acid	Phenolic compounds
BBB permeability	Yes	Yes	Not reported	Yes
CNS	Microglial maturation (*via* FFAR2) Anti-inflammatory (*via* reduced *IL-1b*, *IL-9*, *TNF* expression and NF-kB signaling pathway inhibition in microglia) Anti-depressant (*via* HDAC inhibition) Promotes learning and memory (*via* HDAC inhibition) Attenuates social deficits Modulates levels of neurotransmitters and neurotrophic factors Anti-oxidant Anti-apoptotic (*via FOXM1*, *BRCA2* and *p53* expression) Maintains integrity of the BBB (*via* NFE2L2, tight junction occluding expression) Promotes remyelination	Promotes neuritogenesis	Improves behavioral deficits and reduces infarct volume Limits BBB damage (*via* MMP-2 and MMP-9 inhibition) Anti-oxidant Anti-depressant	Promotes fetal thalamocortical axogenesis
Other potential targets	Maintains intestinal barrier integrity (*via* AMPK, *MUC2* expression, *via* STAT3 signaling) Gut hormonal regulation (*via* FFAR2/3; *GLP1*, *PYY* and leptin expression) Influences gastrointestinal motility (*via* SCFA receptors) Promotes intestinal gluconeogenesis (*via* FFAR3 and *G6PC* and *PCK1* expression) Release of 5-HT (*via* tryptophan 5-hydroxylase 1 expression) Anti-cancer Anti-oxidant (*via* SOD and CAT expression) Modulates immune function and anti-inflammatory (*via* FFAR2/3, HCAR 2/GPR109A and HDAC inhibition)	Cardioprotective effect Anti-inflammatory (*via* inhibition NF-kB) Anti-oxidant	Anti-oxidant (*via* eNOS activity) Inhibits amyloid formation of human islet amyloid polypeptide	Inhibits bone resorption and regulation of osteoclastogenesis (*via* HCAR2/GPR109A)
References	(64–70)	(71–74)	(75–79)	(80–84)

Metabolites	Urolithin A	Enterolactones	Resveratrol-derived metabolites
Food sources	Ellagic acid	Lignans	Resveratrol
BBB permeability	Yes	Yes	Not reported
CNS	Protects against ischemic brain injury (*via* autophagy and ER stress suppression) Prevents learning and memory deficits and reduces Aβ levels (*via* autophagy and Sirt1) Anti-inflammatory (*via* AhR and NF-kB pathway inhibition) Improves associative memory and neuronal survival Reduces white matter demyelination	Anti-inflammatory and anti-oxidant	
Other potential targets	Alleviates myocardial ischemia/reperfusion injury (*via* PI3K/Akt pathway) Anti-cancer (*via* estrogen receptors) Anti-inflammatory (*via* AhR and NF-kB pathway inhibition) Anti-oxidant Increases mitophagy (*via* PINK1-Parkin) and mitochondrial function Increases skeletal muscle function (*via* Sirtuin1 and Pgc1a)	Anti-cancer and anti-metastatic (*via* IGF1/IGF-1R system inhibition and *via* estrogen receptors) Inhibit Akt signaling pathway Inhibit carbonic anhydrase and acetylcholinesterase Anti-diabetic (*via* AMPK signaling)	Anti-cancer (anti-estrogenic activity) Anti-oxidant Anti-inflammatory (inhibit COX (−2) and QR2 enzymes)
References	(85–95)	(96–101)	(102–104)

Metabolites	Equol	CMPF	CLA/CLNA	HYA
Food sources	Daidzein	Fatty fish (furan acid and/or long chain *n*–3 PUFAs)	Linoleic and linolenic acid	linoleic acid
BBB permeability	Yes	Not reported	Yes	Not reported
CNS	Neuroprotective (*via* estrogen receptors, NO production inhibition in astrocytes *via* GPR30) Anti-inflammatory (inhibition NF-kB pathway in microglia)		Neuroprotective Anti-depressant (*via* Nrf2 pathway) Anti-oxidant Anxiolytic and reduction lipid peroxidation in the offspring	Neuroprotective (*via* ERK)
Other potential targets	Estrogenic activity Anti-oxidant (*via* ER receptors, modulates NO release *via* Akt, attenuates ER stress *via* Nrf2) Anti-cancer (*via* Akt pathway, *via* mitophagy) Cardioprotective effect (improves arterial stiffness) Anti-diabetic (*via* cAMP pathway and preventing GLP-1 secretion)	Prevents steatosis (*via* ACC inhibition and induction of FGF21) Induces β cell dysfunction	Anti-cancer (*via* PPAR, inhibition COX-1) Inhibits adipogenesis (*via* PPAR) Anti-inflammatory (*via* NF-kB) Mediates insulin release (FFA1/GPR40 activation)	Anti-inflammatory (*via* GPR40)
References	(105–112)	(50, 113)	(114–122)	(123, 124)

*Dietary fibers* are complex carbohydrates found in fruits, vegetables, legumes and whole grain. The soluble fibers are not digested by human enzymes but are fermented by the gut microbiota (132). Observational studies have shown that dietary fiber intake is associated with better cognitive performance in children aged 7–9 years old (133) and in elderly (134, 135). High consumers of fibers have a lower risk to develop anxiety, psychological distress or depression (7, 136). Dietary fibers intervention with inulin (found notably in onion, chicory, banana, garlic, Jerusalem artichoke and leek) in alcohol use disorder patients improves sociability score and increases circulating levels of brain derived neurotrophic factor (BDNF) during withdrawal (137). The gut microbiota fermentation of soluble dietary fiber stimulates the production of SCFAs that reach the bloodstream and influence the function of several organs (64). If SCFA can cross the blood brain barrier when administered systemically in different animal models, the brain uptake in humans seems to be minimal (64). In rodents, a fiber-deprived diet impairs cognition and SCFA blood levels (138). Moreover, the genetic ablation of GPR 41 and 43 which are SCFA receptors leads to cognitive alteration (138). In a mice model of Alzheimer’s disease (AD), fructans stimulates the release of SCFA and improves cognition, an effect lost upon antibiotic treatment (139). Besides inulin and fructans, other fibers like pectins (found notably in apple) or arabinoxylan (found in cereal) has been found to improve cognition and depressive-like behavior in rodents (140, 141). The SCFAs propionate and butyrate alter astrocyte metabolism which could be involved in cognitive alteration (139). SCFA dietary supplementation also attenuates anhedonia, intestinal permeability and stress responsiveness in chronically stressed mice (142). In mice, inulin-rich diet and butyrate protect against aging-associated brain inflammation (143). SCFAs are essential for the maturation of microglia, which are brain resident immune cells (67). In particular, acetate improves microglia transcriptomic signature and mitochondrial activity that is altered in germ-free mice (66). This was associated with a lower engulfment of amyloid deposits in a mice model of AD (66). In healthy men, supplementation with SCFA attenuates the cortisol response to psychosocial stress (144). Levels of SCFA actually reaching the brain are quite low (64), thus further studies clarifying the mechanisms through which SCFAs modulate neurobiological processes are required. Moreover, comparison of the effectiveness of the different type of soluble fiber in counteracting emotion or cognitive disturbances in preclinical or clinical settings would be interesting. It could be useful to decipher the mechanisms through which dietary fiber may improve brain health besides their stimulating effect on SCFA production.

Medi-diet or MIND diets are particularly rich in fruits, vegetables, olive oil, wine which are particularly rich in *polyphenols*. Numerous clinical studies and meta-analysis report that polyphenols and polyphenol rich-food dietary interventions have both beneficial effects on cognition and/or emotional symptoms in healthy or diseased (obese, depressed, cognitively impaired population) cohorts (9, 145–155). Despite the efficiency of polyphenols on several psychological and cognitive dimensions, more work is needed to precisely determine how it affects brain activity (156). Interestingly, dietary polyphenols are poorly absorbed and are metabolized through a collaboration between host and its gut microbiota improving their absorption and their ability to cross the blood–brain barrier thus resulting in an increased bioactivity (BBB) (99, 157, 158). Of note, the structure of the polyphenols and especially the degree of polymerization has been shown to modulate the production of GDM from polyphenols (159).

Polyphenols encompass two families (flavonoids and non-flavonoids) including several subgroups such as anthocyanins, flavanols, phenolic acids or lignans among others (58). Dietary supplementation containing high levels of flavonoids improves cognitive performances and increases serum BDNF in adult men aged from 26 to 70 years old and aged women (160). Interestingly, polyphenols are potent modulators of the gut microbiota composition. In turn, microbiota is essential to metabolize polyphenolic compounds into bioactive molecules (161). Actually, polyphenols are poorly absorbed in the upper part of the gastrointestinal tract and 90% reach the colon where the gut microbiota metabolizes them (162, 163). Polyphenols affect gut microbiota composition through their prebiotic effect. They stimulate the growth of health-promoting bacteria such as *Akkermansia muciniphila*, *Faecalibacterium prausnitzii*, *Bifidobacteria* and *Lactobacilli* while they inhibit the growth of bacteria with reported detrimental effects through antimicrobial activity, leading to the concept of “duplibiotics” (58). Catechins and epicatechins, which are flavanols enriched in cocoa and tea are metabolized by the gut microbiota (161, 164) into dihydroxyphenyl-γ-valerolactones (DPVL) which ultimately reach the brain and stimulate neuritogenesis (71, 73). The chlorogenic acid, one of the main bioactive compounds of coffee, is converted into caffeic acid which is absorbed or metabolized into dihydrocaffeic acid (DHCA) by gut bacteria (59). DHCA improves behavioral deficits and reduces infarct volume in the brain of rat with cerebral ischemia (72). It also decreases the production of interleukin (IL)-6 and counteracts depressive-like behavior in a mice model of stress (79). Hippurate is a CM product of phenolic compound first metabolized into benzoate by gut microbiota and further conjugated to glycine in the liver (165). Hippurate levels increases with intake of fruit and vegetable (165–168) and gut microbiota diversity (167). In a mice model of obesity, its peripheral administration improves metabolic health (169). It has been reported that hippurate reaches the brain (81, 82) and promotes neuritogenesis in the thalamo-cortical pathway during development (82). As previously reported, urolithin A is produced by gut bacteria from hydrolysable tannin, especially gut-derived ellagic acid (91, 170). Pomegranate, which is particularly rich in ellagic acid has been shown to be protective in several rodent models of neurodegeneration (171). Urolithin A is among the pomegranate metabolites with the highest ability to cross the BBB, making this GDM a good candidate for pomegranate’ neuroprotective effects (95). In addition, urolithin A alleviates neuroinflammation *in vitro* (89), in rodent models of AD and multiple sclerosis (91, 92, 170) and modulates microglia phagocytosis activity and mitophagy as well as AMP-activated protein kinase (AMPK) and the nuclear factor-kappa B (NFκB) signaling (88). Urolithin A activity is mediated by its binding to Aryl Hydrocarbon Receptor (AhR) (88). The enterolactones are produced through the metabolization of lignans by gut bacteria including *Ruminococcus* species (172). These compounds exert anticholinesterase activity and thus have been proposed as candidates to tackle neurodegenerative disorders like AD (172). Even if the molecular mechanisms remain unknown, preclinical findings have shown that enterolactones are neuroprotective in AD and Parkinson disease (PD) animal models (98, 173). Dihydroresveratrol (DHR), lunularin (LUN) and 3,4′–dihydroxy-trans-stilbene have been identified as gut microbiota-derived metabolites of resveratrol (53) with some having more potent anti-inflammatory effects than resveratrol (102). Interestingly, these GDM and the resveratrol itself can be transported within extracellular vesicles which represent a very interesting pathway to study in order to better understand how GDM and CM can influence the brain (62). The isoflavones like daidzein is metabolized by gut bacteria into equol that exhibits, as its precursor, neuroprotective effects *in vitro* possibly through its ability to activate oestrogen receptors (106, 174). Overall, a large amount of polyphenols-derived GDM and CM deserve more attention as their bioactive properties can be responsible for the widely recognized health-promoting effects of polyphenols (58, 175). Regarding the neurobiological/behavioral response toward food intervention, taking into account the interindividual differences in both gut microbiota and host metabolism of nutrients like polyphenols is crucial. Indeed, individual may not be able to produce polyphenols-derived metabolites for several reasons (lacking the appropriate bacteria or disturbances in phase II metabolites producing enzymes) which may introduce a bias in the interpretation of the efficacy of nutritional intervention. Of note, the structure of the polyphenols and especially the degree of polymerization has been shown to modulate the production of GDM from polyphenols which underline the importance of the food sources and the choice of the compounds selected in nutritional intervention (159). Thus, studying GDM and CM would allow to select individuals that may benefit from the nutritional supplementation, to select the better compounds and to adapt the strategy by using symbiotic or postbiotic approach in individual who are not able to produce the GDM/CM of interest.

Several type of GDM and CM can be produced upon n-3 *PUFA* consumption (113, 124, 176). The 3-carboxy-4-methyl-5-propyl-2-furanpropanoic acid (CMPF) is a GDM produced in response to fatty fish intake or dietary supplementation (113, 177, 178). This GDM has been associated with a slower cognitive decline in middle-aged men (179). Its bioactive potential has been shown especially in the context of steatosis where it mediates the beneficial effects of *n*–3 PUFA (113). The gut microbiota also produces some conjugated compounds like the conjugated linoleic acid (CLA) or the conjugated linolenic acid (CLNA) from *n*–6 and *n*–3 PUFA precursors, respectively, (176). When administered through the diet, CLA alleviates several markers of brain ageing in a mice model of lupus (114, 115). As a result, CLA improves synaptic markers and BDNF decrease as well as acetylcholine esterase activity and oxidative stress in the brain of aged mice (114). CLA administered to mice during pregnancy and lactation triggers anxiolytic and antioxidant effects in offspring (119). Of note, the brain effect of CNLA can involve changes in the level of long-chain PUFAs in the brain which is known to influence several neurobiological mechanisms (8, 182–185). Indeed, several studies have shown that these metabolites can change the level of long-chain PUFA in the liver and in the brain (114, 118). Finally, the 10-hydroxy-cis-12-octadecenoic acid (HYA) which is produced by some *Lactobacillus* has been shown to protect from obesity related alterations like insulin-resistance and adipose tissue inflammation (124). It also attenuates neuroinflammatory processes by inhibiting ERK phosphorylation in activated microglia *in vitro* (123).

## 4. Concluding remarks and future challenges

Although the molecular mechanisms are largely unexplained, several GDM/CM seem to be neuroactive compounds. Thus, exploring further their role in the crosstalk between nutrition, gut microbiota and brain function may help to improve nutritional intervention by giving new possibilities to intervene in people who are not able to respond to food or nutrients-based supplementation. Several challenges arise from the complex relationship between the GDM/CM and nutrition, gut microbiota, clinical status as well as their pleiotropic effect. Indeed, GDM/CM production is controlled by several parameters (22, 23). A first challenge is therefore to unravel the mechanisms through which GDM and CM affect brain function. As discussed in this review, understanding the mechanisms involved in the inter-individual differences in the ability to produce GDM/CM in humans will allow to better select the target population that would benefit from nutritional intervention. For this, it is necessary to study the production of GDM/CM of interest in response to dietary intake of specific healthy food (nuts, fish, dark leaf vegetables …) or a specific precursor (lignans, tannins, fibers, PUFA) in healthy and diseased populations. To confirm that GDM/CM may mediate the effect of nutrition and gut microbiota on brain function, more mechanistic studies are needed. They should aim at (1) decipher how GDM/CM reach the brain, (2) test the specificity of the effect of these molecules on different brain structures and brain cell types, and (3) elucidate their receptors and molecular targets. To achieve these goals, it is necessary to combine dietary habits assessments and circulating GDM/CM measurements especially in clinical studies. The use of predictive models may help to select a pool of metabolites associated with beneficial effects on neurobiological process or behavior rather than a single GDM. It would allow to design innovative intervention with postbiotics alone or in combination which efficiency could then be tested in preclinical models. *In vitro*, the use of fermentation models (159, 184) can help to decipher the bacteria and the molecular machinery involved in the production of GDM.

## Author contributions

QL, LP-P, and SL wrote or contribute to the writing of the manuscript. QL created the figures and LP-P created the table. All authors contributed to the article and approved the submitted version.

## Funding

SL was supported by the Chaire d’excellence Région Nouvelle Aquitaine ExoMarquAge (13059720–13062120), the Région Nouvelle Aquitaine Autisme (2019-1R3M08 AUTISME), the JPND SOLID (ANR-21-JPW2-0004-05), the RRI Food4BrainHealth, INRAE. QL and SL were supported by the ERA-NET NEURON 2019 Translational Biomarkers Grant Gut2Behave (ANR-19-NEUR-0003-03).

## Conflict of interest

The authors declare that the research was conducted in the absence of any commercial or financial relationships that could be construed as a potential conflict of interest.

## Publisher’s note

All claims expressed in this article are solely those of the authors and do not necessarily represent those of their affiliated organizations, or those of the publisher, the editors and the reviewers. Any product that may be evaluated in this article, or claim that may be made by its manufacturer, is not guaranteed or endorsed by the publisher.
